# The regulatory process and practical significance of non-coding RNA in the dissemination of prostate cancer to the skeletal system

**DOI:** 10.3389/fonc.2024.1358422

**Published:** 2024-03-21

**Authors:** Hui Sang, Luxi Li, Qiang Zhao, Yulin Liu, Jinbo Hu, Peng Niu, Zhenming Hao, Keqiang Chai

**Affiliations:** Department of Urology, The Third Affiliated Hospital of Gansu University of Traditional Chinese Medicine, Baiyin, China

**Keywords:** prostate cancer, bone metastasis, non-coding RNA, micro RNA, long non-coding RNA, circular RNA, signaling pathway, targeted therapy

## Abstract

Prostate cancer is a major contributor to male cancer-related mortality globally. It has a particular affinity for the skeletal system with metastasis to bones seriously impacting prognosis. The identification of prostate cancer biomarkers can significantly enhance diagnosis and patient monitoring. Research has found that cancer and metastases exhibit abnormal expression of numerous non-coding RNA. Some of these RNA facilitate prostate cancer bone metastasis by activating downstream signaling pathways, while others inhibit this process. Elucidating the functional processes of non-coding RNA in prostate cancer bone metastasis will likely lead to innovative treatment strategies for this malignant condition. In this review, the mechanistic role of the various RNA in prostate cancer is examined. Our goal is to provide a new avenue of approach to the diagnosis and treatment of bone metastasis in this cancer.

## Introduction

Among the leading causes of cancer deaths, prostate cancer (PCa) comes in fifth globally, and is the second most common cancer in men (1). The spread of cancer is the primary reason for mortality in PCa. PCa has a strong affinity for bone, with 80% of metastases involving various bones such as the hip, spine and pelvis (2, 3). About half of men with androgen-responsive advanced PCa are likely to experience bone metastasis within two years. Patients without bone metastasis have a 5-year survival rate of 56%, whereas those with have a significantly lower rate of 3% (4). Thus, patients with bone metastasis have a poor prognosis, and are associated with significant mortality. PCa bone metastasis presents a thorny problem in patient management. Reported studies showed that bone tissue-related protein molecules - WISP-1, BHLHE22, KLF5, CXCL12/CXCR4, GDF15 - regulate the adhesion and colonization of PCa cells in bone tissue (5–9). In addition, molecular signaling interactions between cancer cells and the marrow environment lead to preferential establishment of metastatic PCa cells in bone (10–13). The effect of these molecules on PCa bone metastasis provides a basis for the development of targeted therapies (14). In recent years, researchers have also tried to explore the mechanism of non-coding RNA (ncRNA) in the process of prostate cancer bone metastasis.

Recent research has shown that ncRNA have a major impact on tumor progression, spread, and drug resistance (15, 16). Numerous investigations have been undertaken to study the mechanism by which ncRNA control these processes. The ncRNA can be divided into categories based on their length, shape and localization. In cancer, three main categories are known: micro RNA (miRNA), long non-coding RNA (lncRNA), and circular RNA (circRNA), with each playing distinct roles (17). miRNA, which are short RNA molecules of about 20 nucleotides, bind to matching sequences in target mRNA to initiate degradation through the RNA-induced silencing complex (18). lncRNA are more than 200 nucleotides in length, and circRNA are of hundreds to thousands of nucleotides long. They can originate from exons, introns, intergenic regions, or the 5’ and 3’ untranslated regions. These molecules can adopt intricate secondary structures enabling their interaction with DNA, RNA, and proteins (19). Various mechanisms are utilized by lncRNA and circRNA to modulate gene expression. By functioning as lure for miRNA, they can short circuit the degradation of targeted mRNA, and tweak the interaction between transcription factors and promoters (20). They can serve as scaffolds to affect interactions between proteins and downstream signaling pathways. Recent studies have also shown that lncRNA and circRNA are involved in epigenetic modification of chromatin to regulate gene expression. In addition, circRNA can function to encode proteins (21). A substantial amount of evidence has shown the influence of ncRNA on multiple tumors. These molecules can behave as oncogenes or tumor suppressor genes in the initiation and progression of cancer (22).

**Figure 1:**
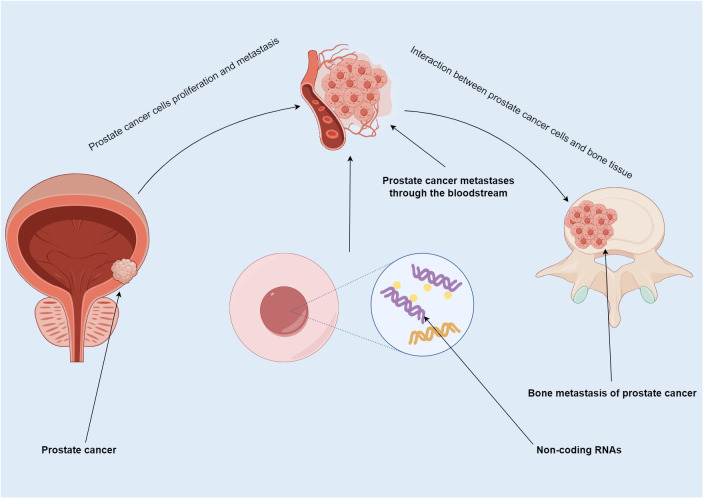
Summary map of non-coding RNA involvement in PCa bone metastasis. PCa usually spreads to bone. In this process, ncRNA not only participate in the proliferation and metastasis of PCa, but also regulate the progression of tumor cells in bone.

In PCa metastasis, ncRNA not only participate in proliferation and spread but also regulate progression of tumor cells in bone (23–25) (**Figure 1**). More than half of miRNA are localized in genomic regions that affect the expression levels of pro-transfer genes by targeting their transcribed mRNA (26, 27). miRNA expression found in PCa is highly abnormal, and miRNA promote or inhibit bone metastasis by regulating invasion and migration (28–30). Secondly, the biological function of miRNA in the tumor microenvironment requires the diffusion of exosomes. Exosomes are tiny vesicles containing multiple miRNA (31, 32). They function as intercellular messengers facilitating the transfer of miRNA from one cell to another. Studies have reported that exosomic miRNA could have an impact on the microenvironment of bone metastasis through their action in signaling (33–36). Some lncRNA promote tumor progression in PCa. For example, lncRNA AC245100.4 was shown to promote progression via the miRNA-145-5p/RBBP5 axis (37), and lncRNA TUG1 via miRNA-128-3p/YES1 (38). Dysregulated lncRNAs expression has been shown to exert a role in the disease course of many types of malignancies. More recent research emphasized the significance of lncRNA as critical mediators of signal transduction in cancer (39). The disruption of certain lncRNA targets was linked to the stage and prognosis of cancer (40–42). Mutant expression of lncRNA in PCa was reported regarding its prognostic effect and specific expression patterns in cancer subtypes (43). Lastly, circRNAs are circular molecules formed by transsplicing, which are highly stable and can be detected in body fluids. They are involved in many important molecular regulatory mechanisms (44), and may have unique advantages in diagnosis, treatment, monitoring, and prognosis. Studies have found a role of circRNA in the onset and progression of PCa. By snaring miRNA-646 and enhancing TGF1 production, circRNA-51217 increases TGF1/P-SMAD signaling in PCa cell invasion (45). Through upregulating XIAP, the hsa-circ-0005276/FUS axis promotes PCa cell proliferation, migration, epithelial-to-mesenchymal transition (EMT) (46), and therapeutic resistance (47). In sum, ncRNA may serve as therapeutic targets, especially for patients with bone metastases. (NB: transition between the first two paragraphs is problematic, please correct the reference numbering. I made it so that the flow is smoother: miRNA first, then lncRNA, and last circRNA).

**Figure 2:**
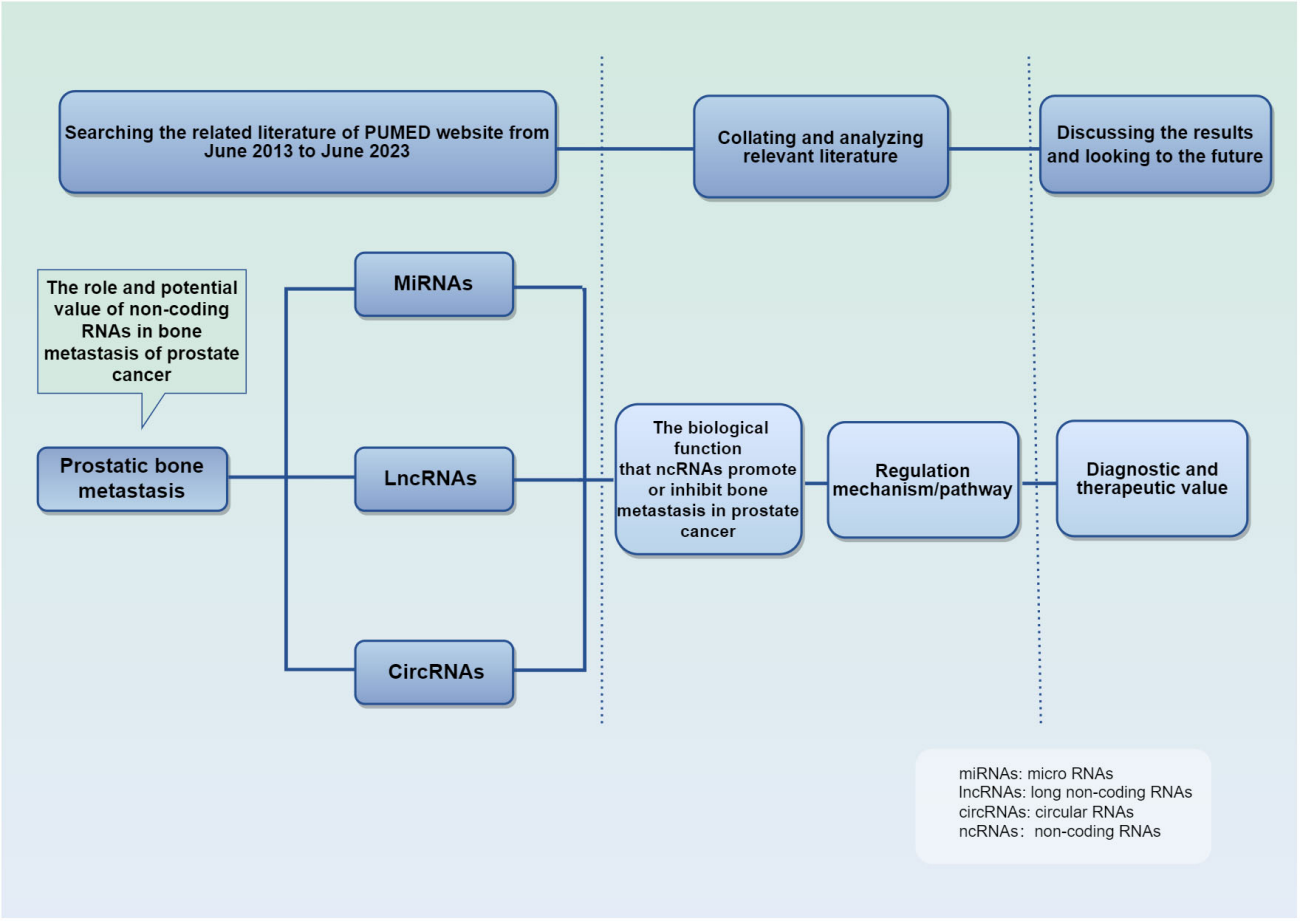
Schematic conceptual framework for the research questions and methods. It details the purpose and methods of the studies.

We searched on PUBMED relevant studies of ncRNA in PCa bone metastasis from June 2013 to June 2023, summarized their mechanism of action and biological function, and discussed their diagnostic and therapeutic value (**Figure 2**). The studies retrieved have confirmed their importance in PCa bone metastasis. Parenthetically, telomerase RNA is also found highly expressed in PCa, and is involved in tumor progression (48–50). This review summarizes the unique mechanism of ncRNA in the development of bone metastasis, suggests their utility despite current limitations.

## Association of miRNA with PCa bone metastasis

**Figure 3:**
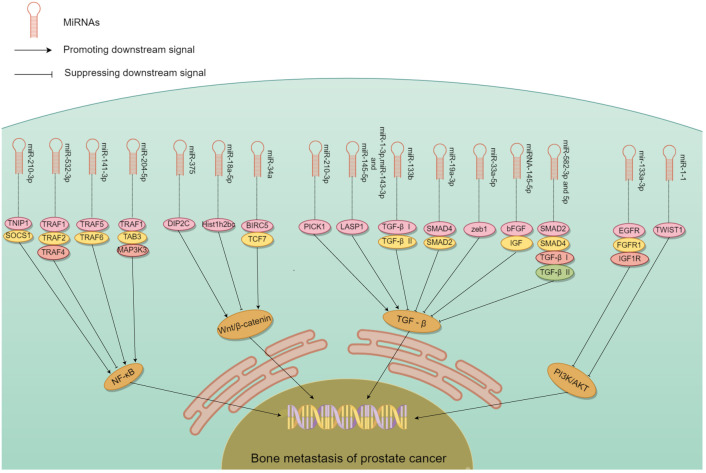
The mechanism of different miRNA involved in PCa bone metastasis. Many miRNA act on downstream molecules to regulate PCa bone metastasis through NF-κB, Wnt, TGF-β, PI3K/AKT and other signaling pathways. Some miRNA play a role in promoting PCa bone metastasis, while others play an inhibitory role.

The role of miRNAs in tumor metastasis has been documented in various cancer types, in particular, PCa bone metastasis (51–53). Some miRNA promote bone metastasis, while some inhibit (**Table 1**). The controlling role either stimulates or impedes signaling by nuclear transcription factor-κB (NF-κB), Wnt, transforming growth factor β (TGF-β), PI3K/AKT, and others (**Figure 3**). miRNA promoting bone metastasis include miR-210-3p, miR-375, miR-18a-5p, miR-210-3p, miR-1-3p/143-3p/145-5p, miR-409-3p/-5p, and miR-378a-3p. miRNA inhibiting bone metastasis include miR-532-3p, miR-141-3p, miR-204-5p, miR-34a, miR-133b and miR-19a - 3p, miR-33a-5p, miR-582-3p/5p, miRNA-145-5p, miR-1-1, miR-133a-3p, miR-466 (**Table 1**).

**Table 1:** Aberrantly expressed miRNA regulate bone metastasis in PCa.

microRNAs	Expression in tumour	Regulation Mechanism	Related pathway	Experiment System	Ref.
miR-409-3p/-5p	upregulated	miR-409-3p/-5p promotes tumor growth, EMT, and bone metastasis	EMT	Clinical sample analysis, *in vitro* and *in vivo* studies	S. Josson et al. (54) 2014
miR-34a	Downregulated	miR-34a targets TCF7 and BIRC5	Ras and Wnt signaling pathways	Clinical sample analysis, *in vitro* and *in vivo* studies	W. Y. Chen et al. (55) 2015
miR-1	Downregulated	miR-1-1 acts directly on TWIST1	PI3K/AKT signaling pathway	Clinical sample analysis, *in vitro* and *in vivo* studies	Y. S. Chang et al. (56) 2015
miR-466	Downregulated	miR-466 works by directly targeting the bone-associated transcription factor RUNX2	RUNX2 signaling pathway	Clinical sample analysis, bioinformation mining, *in vitro* and *in vivo* research	M. Colden et al. (57) 2017
miR-210-3p	upregulated	miR-210-3p acts on PICK1	TGF-β signaling pathway	Clinical sample analysis, *in vitro* and *in vivo* studies	Y. Dai et al. (58) 2017
miR-141-3p	Downregulated	miR-141-3p directly targets tumor necrosis factor receptor-associated factors 5(TRAF5) and 6 (TRAF6) to inhibit the activation of the NF-κB signaling pathway	NF-κB signaling pathway	Clinical sample analysis, bioinformation mining, *in vitro* and *in vivo* research	S. Huang et al. (59) 2017
miR-210-3p	upregulated	miR-210-3p maintains sustained activation of NF-κB signaling by targeting negative regulators of NF-κB signaling TNIP1 and SOCS1	NF-κB signaling pathway	Clinical sample analysis, *in vitro* and *in vivo* studies	D. Ren et al. (60) 2017
miR-133b	Downregulated	miR-133b inhibits the activity of the TGF-β signaling pathway by directly targeting TGF-β receptors I and II	TGF-β signaling pathway	Clinical sample analysis, *in vitro* and *in vivo* studies	S. Huang et al. (61) 2018
miR-133a-3p	Downregulated	miR-133a-3p inhibits the PI3K/AKT signaling pathway by directly targeting EGFR, FGFR1, IGF1R, and MET	PI3K/AKT signaling pathway	Clinical sample analysis, *in vitro* and *in vivo* studies	Y. Tang et al. (62) 2018
miR - 19a - 3p	Downregulated	miR-19a-3p targets SMAD2 and SMAD4, resulting in TGF-β signaling inactivation	TGF – β signaling pathway	Clinical sample analysis, *in vitro* studies	Q. Wa et al. (63) 2018
miR-33a-5p	Downregulated	mir-33a-5p targets zeb1 to inhibit TGF-β signaling	TGF-β signaling pathway	Clinical sample analysis, bioinformation mining, *in vitro* and *in vivo* studies	Y. Dai et al. (64) 2019
miR-582-3p and miR-582-5p	Downregulated	miR-582-3p and miR-582-5p inhibit the TGF-β signaling pathway by targeting SMAD2, SMAD4, TGF-β receptor I (TGFBRI), and TGFBRII	TGF-β signaling pathway	Clinical sample analysis, *in vitro* and *in vivo* studies	S. Huang et al. (65) 2019
miR-204-5p	Downregulated	miR-204-5p targets TRAF1, TAB3, and MAP3K3 to inactivate NF-κB signaling	NF-κB signaling pathway	Clinical sample analysis, *in vitro* studies	Q. Wa et al. (66) 2019
miR-532-3p	Downregulated	miR-532-3p targets tumor necrosis factor receptor-associated factor 1 (TRAF1), TRAF2, and TRAF4 inhibit the activation of the NF-κB signaling pathway	NF-κB signaling pathway	Clinical sample analysis, *in vitro* and *in vivo* studies	Q.Wa et al. (67) 2020
miR-18a-5p	upregulated	miR-18a-5p targeted Hist1h2bc gene, resulting in the up-regulation of Ctnnb1 expression in Wnt/β-catenin signaling pathway.	Wnt/β-catenin signaling pathways	Clinical sample analysis, *in vitro* and *in vivo* studies	F. Zeng et al. (68) 2023
miR-375	upregulated	miR-375 targets DIP2C and activates the Wnt signaling pathway	Wnt signaling pathway	Clinical sample analysis, *in vitro* and *in vivo* studies	Y. Liu et al. (69) 2023
miR-1-3p/143-3p /145-5p	upregulated	miR-1-3p/143-3p/145-5p acts on LASP1	TGF-β signaling pathway	Clinical sample analysis, biological information mining, *in vitro* studies	H. Guo et al. (70) 2023
miR-378a-3p	upregulated	miR-378a-3p activates Dyrk1a/Nfatc1/Angptl2 axis in BMMs	Dyrk1a/Nfatc1/Angptl2 axis	*In vitro* and *in vivo* studies	J. Wang et al. (71) 2022
miRNA-145-5p	Downregulated	miRNA-145-5p inhibited the expression of bFGF, IGF and TGF-β	TGF-β signaling pathway	Clinical sample analysis, *in vitro* studies	B. Luo et al. (72) 2022

### NF-κB signaling pathway

In PCa patients, NF-κB regulates the expression of multiple genes in bone metastasis (73–75). It interacts with ncRNA synergistically in this process relating to tumor progression (76). miR-210-3p was linked to the formation, growth, and spread of tumors. Ren et al. (60) used clinical samples to detect miR-210-3p in PCa bone metastasis and non-bone metastasis, and study its mechanism of action. miR-210-3p maintains sustained NF-κB signaling by targeting its negative regulators TNIP1 (TNF-α-induced protein-3 interaction protein 1) and SOCS1 (cytokine signal transduction inhibitor 1) and leading to cancer cell migration and establishment of bone metastasis. This miRNAs can be a candidate target in the treatment. Whether its action on PCa bone metastasis is specific needs to be confirmed.

miRNA that have an inhibitory role in PCa bone metastasis was found by Wa et al. (67). The level of miR-532-3p was reduced in PCa that had spread to bone. Decreased expression of it showed a strong association with Gleason grade and serum level of prostate-specific antigen. Upregulation of miR-532-3p inhibited bone metastasis of PCa cells *in vivo*. Its overexpression suppressed activation of the NF-κB pathway by specifically targeting TRAF1 (tumor necrosis factor receptor-associated factor 1), TRAF2, and TRAF4 to impede migration and metastasis. Huang et al. (59) similarly found that miR-141-3p suppressed activation of the NF-κB pathway by targeting TRAF5 and TRAF6. Wa et al. (66) reported that miR-204-5p inactivated the NF-κB pathway by simultaneously binding TRAF1, TAB3, and MAP3K3 mRNA. The dual role of miRNA in PCa bone metastasis highlights the potential use of these miRNA as serum biomarkers.

### Wnt/β-catenin signaling pathway

The Wnt/β-catenin pathway is well-studied in many cancers, and is modulated by gene mutations, extracellular inhibitors, and transcriptional cofactors (77). miRNA have a significant impact on PCa bone metastsis by their interaction with Wnt/β-catenin signaling. Liu et al. (69) showed that miR-375 exhibited high levels in PCa, and could be transported to human mesenchymal stem cells (hMSC) via exosomes. miR-375 specifically targeted DIP2C, and enhanced Wnt signaling to facilitate the transformation of hMSC into osteoblasts, which in turn stimulated the growth, infiltration, and motility of PCa cells *in vitro*, as well as tumor progression and osteogenic metastasis *in vivo*. Zeng et al. (68) found that miR-18a-5p was transported to osteoblasts by exosomes derived from PCa cells. It specifically targeted the Hist1h2bc gene, leading to an increase in Ctnnb1 expression in the Wnt/β-catenin pathway. *In vivo* studies have shown that anti-mir-18a-5p can significantly improve bone biomechanics of osteoblastic metastasis and reduce sclerosis. Blocking the exosome transport of miR-18a-5p also ameliorates osteoblastopathy caused by PCa.

Another crucial molecule in bone metastasis is miR-34a, which directly targeted TCF7, a gene linked to Wnt signaling (55). In PCa patient samples, miR-34a levels were negatively correlated with TCF7 expression. Chen et al. (55) demonstrated the role of miR-34a in tumor proliferation by targeting TCF7. miR-34a defects are necessary for the anti-apoptotic effects observed in Ras signaling-activated PCa cells. Furthermore, these defects enhanced tumor cell survival by the activation of Wnt and anti-apoptotic signaling pathways leading to the expression of TCF7 and BIRC5. In Ras-dependent xenotransplantation models, expression of miR-34a outside its normal site hindered the spread of cancer to bone, and reduced cancer cell growth. Thus, some miRNA could be converted into tumor suppressors due to their inhibitory function on bone metastasis.

### TGF-β signaling pathway

Sustained activation of the TGF-β signaling pathway plays an essential role in the development of bone metastasis, which could be affected by miRNA (8, 78). Overexpression of PICK1 could impede invasion and migration of PCa cells *in vitro*, as well as bone metastasis *in vivo*. Dai et al. (58) found that overexpression of miR-210-3p in PCa bone metastases led to downregulation of PICK1 and, as a result, promotion of bone metastasis. Guo et al. (70) found that expression of miR-1-3p, miR-143-3p and miR-145-5p was correlated with bone metastasis. Functional experiments demonstrated that miR-1-3p/143-3p/145-5p promoted the proliferation and migration of PCa cells *in vitro*. LASP1 is the common target of these miRNA, which interacts with TGF-β. *Shouldn’t this belong in the previous paragraph?*

Decrease in other miRNA was linked to PCa spread to bone, while their overexpression suppressed bone metastasis. Huang et al. (61) found that increasing the expression of miR-133b hindered invasion and motility of PCa cells *in vitro* as well as frequency of bone metastasis *in vivo*. miR-133b hindered TGF-β signaling by targeting TGF-β receptors I and II to suppress bone metastasis. Dai et al. (64) found that increasing the expression of miR-33a-5p suppressed EMT, invasive behavior of PCa cells. Conversely, silencing of miR-33a-5p led to promotion of invasive behavior. Elevated zeb1 copy number by mir-33a-5p apparently induced a TGF-β signaling-dependent negative feedback loop. Huang et al. (65) reported that upregulation of miR-582-3p and miR-582-5p also had an inhibitory effect on PCa cells *in vitro* and bone metastasis *in vivo*. Both miR-582-3p and miR-582-5p suppressed the TGF-β pathway by targeting its multiple elements including SMAD2, SMAD4, TGF-β receptor I (TGFBRI), and TGFBRII. Luo et al. (72) showed that miRNA-145-5p had the ability to suppress growth, motility, and invasiveness of bone metastatic PCa cells. Additionally, miRNA-145-5p could suppress the expression of bFGF, IGF, and TGF-β in PC3 cells. miRNA-145-5p negatively regulated EMT, inhibited bone metastasis, and promoted apoptosis of bone metastatic cells. Thus, miRNA could both promote or inhibit cancer via the TGF-β pathway.

### PI3K/AKT signaling pathway

Some miRNA appeared to inhibit bone metastasis by disrupting the PI3K/AKT signaling pathway (79, 80). Tang et al. (62) found that the levels of miR-133a-3p were lower in tumor tissues than nearby normal/benign tissues, particularly in PCa that had spread to the bone. Importantly, mir-133-a-3p overexpression could effectively hinder the development of bone metastasis *in vivo*. This miRNA effectively reduced the spread of PCa to bone by targeting multiple cytokine receptors such as EGFR, FGFR1, IGF1R, and MET. This inhibition of PI3K/AKT signaling plays a crucial role in preventing PCa bone metastasis. Likewise, miR-1 exhibited an inhibiting impact on PCa cells, and its expression was linked to a decrease in the likelihood of metastasis. Chang et al. (56) demonstrated that translocation of EGFR could regulate the transcription of miR-1-1, which then acted directly on TWIST1 expression. Nuclear EGFR functioned as a transcription inhibitor to restrict the tumor-suppressing impact of miR-1, and sustain the oncogenic stimulation of TWIST1 resulting ultimately in enhanced bone metastasis. These studies validated that miRNA could hinder the PI3K/AKT pathway in PCa bone metastasis.

### Other pathways (?)

Additional miRNA in the spread of PCa to bone were also identified. Josson et al. (54) showed that embryonic stem cell miR-409-3p/-5p was expressed in elevated levels in PCa bone metastatic cell lines. miR-409-3p/-5p showed a significant impact on PCa by facilitating tumor growth, promoting EMT and bone metastasis. The role of extracellular vesicles (EV) in the creation of a pre-metastasis microenvironment is also significant. In PCa bone metastasis, the release of miR-378a-3p from cancer cells via EV was increased (71). This release maintained a low concentration of miR-378a-3p inside the cells, thereby promoting proliferation and EMT. Activation of the Dyrk1a/Nfatc1/Angptl2 axis by miR-378-a-3p promoted PCa bone metastasis. By using multimodal strategy analysis with xenograft models, miR-466 was shown to inhibit orthotopic tumor growth and spontaneous bone metastasis (57). This inhibition was mediated by miR-466 through targeting of the bone-related transcription factor RUNX2.

## Association of lncRNA with PCa bone metastasis

**Figure 4:**
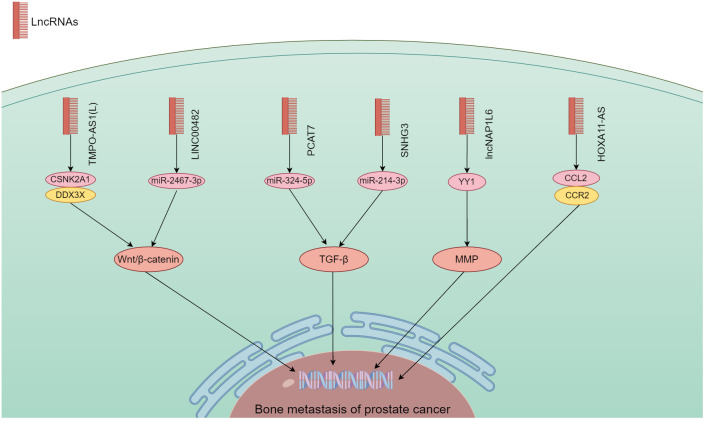
The mechanism of different lncRNA responsible in PCa bone metastasis. The lncRNA carry out their biological functions by utilizing the Wnt/β-catenin, TGF-β, MMP, and chemokine signaling pathways.

lncRNA have the ability to bind DNA, RNA and protein, and play a role in cell proliferation, migration, differentiation, as well as angiogenesis. Abnormal expression of lncRNA is common in a variety of cancers (41). They have a significant impact on the onset and progression of various malignancies (81, 82). According to the findings in **Table 2**, a large quantity of lncRNA was shown to involve in PCa bone metastasis. Their biological functions utilized Wnt/β-catenin, TGF-β, MMP, and chemokine signaling pathways (**Figure 4**).

**Table 2:** Aberrantly expressed lncRNA regulate bone metastasis in PCa.

LncRNAs	Expression in tumour	Regulation Mechanism	Related pathway	Experiment System/ Participants	Ref.
PCAT7	upregulated	PCAT7 up-regulates TGFBR1 expression by adsorption of miR-324-5p, thereby activating TGF-β/SMAD signaling pathway.	TGF-β/SMAD signaling pathway	Clinical sample analysis, biological information mining, *in vitro* studies	C. Lang et al. (83) 2020
KCNQ1OT1	upregulated	KCNQ1OT1 up-regulates CHI3L1 and promotes PCa progression through competitive binding to miR-211-5p.	lncRNA KCNQ1OT1/miR-211-5p/ CHI3L1 axis	Clinical sample analysis, *in vitro* and *in vivo* studies	H. Hao et al. (84) 2021
NORAD	upregulated	NORAD promotes the expression of PKM2 by mediating miR-541-3p	NORAD/miR-541-3p/PKM2 axis	Clinical sample analysis, biological information mining, *in vitro* studies	C. Y. Hu et al. (85) 2021
HOXA11-AS and HOXB13	upregulated	After binding to HOXA11-AS, HOXB13 directly regulates the integrin-binding salivary protein (IBSP) promoter	CCL2/CCR2 and integrin signaling pathways	*In vitro* study	A. Misawa et al. (86) 2021
NEAT1	upregulated	NEAT1 competitively binds miR-205-5p via the SFPQ/PTBP2 axis.	SFPQ/PTBP2 axis	*In vitro* and *in vivo* studies	C. Mo et al. (87) 2021
lncNAP1L6	upregulated	lncNAP1L6 interacts with YY1 to promote transcription of MMP2 and MMP9	MMP signaling pathway	*In vitro* study	Zheng Y et al. (88) 2023
TMPO-AS1	upregulated	TMPO - AS1 enhance CSNK2A1 and DDX3X interaction, activation of Wnt/beta - catenin signaling pathway	Wnt/β-catenin signaling pathways	*In vitro* and *in vivo* studies	M. Wang et al. (89) 2023
LINC00482	upregulated	LINC00482 binds to miR-2467-3p and activates the Wnt/β-catenin signaling pathway	Wnt/β-catenin signaling pathways	*In vitro* and *in vivo* studies	S. Liao et al. (90) 2023
SNHG3	upregulated	SNHG3 enhances TGFBR1 expression and activates TGF-β signaling pathway by targeting miR-214-3p.	TGF-βsignaling pathway	*In vitro* and *in vivo* studies	X Xi et al. (91) 2022

### Wnt/β-catenin signaling pathway

lncRNA have a regulatory function in the development, spread, and drug resistance of human malignancies (92, 93). By modulating different signaling pathways, lncRNA exert their influence on cancer cells (94). Wang et al. (89) found that an extended version of TPMP-AS1(L) exhibited increased expression in PCa tissues with bone metastasis. An association was noted between overexpression of TPMP-AS1(L) and clinicopathological characteristics plus patient survival. TPMO-AS1(L) could serve as a framework for the association between casein kinase 2α1 (CSNK2A1) and DEAD-box helicase 3 X-linked (DDX3X) leading to activation of the Wnt/β-catenin pathway, and fostering the myelodysplasia of PCa. Liao et al. (90) analyzed the TCGA database set of PCa for the expression of LINC00482, and used real-time quantitative PCR to verify its expression levels in PCa and PCa bone metastases. The biological function of LINC00482 *in vitro* was shown by CCK-8, clone formation. This molecule exhibited notable expression in PCa with bone metastasis, and a correlation with PCa progression. Its silencing inhibited the functioning of PCa cells. LINC00482 had the potential to compete against miR-2467-3p leading to the activation of Wnt/β-catenin.

### TGFβ signaling pathway

There is growing evidence that lncRNAs are involved in TGF-β-driven cancer progression, highly disease-specific, and ideal targets for therapeutic development (95). lncRNA PCAT7 was found associated with bone metastasis through analysis of TCGA data. It showed elevated expression in PCa with bone metastasis (83). Increased expression of PCAT7 facilitated the *in vivo* spread of PCa to bone, as well as migration, invasion, and EMT of PCa cells *in vitro*. It up-regulated TGFBR1 expression by uptake of miR-324-5p to activate TGF-β/SMAD signaling. Disrupting the continuous activation loop of PCAT7 and TGF-β signaling could potentially provide a therapeutic approach in treating bone metastasis. Xi et al. (91) found that SNHG3 expression in PCa bone metastasis was considerably higher than non-metastatic PCa and para-cancerous tissues. PCa patients who exhibited high levels of SNHG3 were likely to have advanced clinicopathology and unfavorable prognosis. By targeting miR-214-3p, SNHG3 boosted the expression of TGFBR1 and triggered TGF-β signaling. It showcased a novel function for the SNHG3/miR-214-3p/TGF-β pathway in the proliferation of tumors and the spread of cancer to bone in PCa.

### MMP and chemokine signaling pathways

lncRNA could also promote bone metastasis by regulating MMP and chemokine signaling. Zheng et al. (88) elucidated the effect of lncNAP1L6 on PCa progression regarding its possible regulatory mechanism. PCa cells exhibited an increase in lncNAP1L6 expression. Cancer cell functions were enhanced by its overexpression in functional testing. Mechanistic experiments demonstrated lncNAP1L6 to interact with YY1 promotion of MMP2 and MMP9 transcription, and activate their signaling. Misawa et al. (86) found that invasion and proliferation of PC3 cells were enhanced by overexpression of HOXA11-AS, which was similar to box A11 antisense RNA, in bone metastatic cell lines. HOXA11-AS appeared to regulate the bone metastasis-associated CCL2/CCR2 signaling pathway in PC3 cells and SaOS2 osteoblasts. In PCa, the HOXB13/HOXA11-AS axis also controlled the integrin subunits specific to bone metastasis.

Other lncRNA that could contribute to the spread of PCa to bone include a complex of KCNQ1OT1/miR-211-5p/CHI3L1 (84). In PCa, the expression of KCNQ1OT1 was increased. Down-regulation of its expression inhibited the functioning of PCa cells. KCNQ1OT1, functioning as a competing endogenous RNA, enhanced the expression of CHI3L1, and facilitated PCa progression by binding to miR-211-5p. Mo et al. (87) investigated the physiological mechanism by which NEAT1-encapsulated exosomes affected PCa progression. NEAT1, carried by exosomes derived from PCa, competitively attached to miR-205-5p via SFPQ/PTBP2 to increase RUNX2 expression *in vitro* and *in vivo*, thereby enhancing the osteogenic differentiation of hBMSC. This finding suggested a promising therapeutic target for promoting osteogenic differentiation in hBMSC affected by PCa. Our understanding of how lncRNA affect PCa bone metastasis will create prospects for better management of this disease.

## Association of circRNA with PCa bone metastasis

circRNA are ncRNA formed by back-splicing. Due to its distinct composition and quality, it has great potential in diagnosis, management, and prognosis of cancerous growths. Differential expression of these molecules was conducted with circRNA microarrays in normal prostate epithelial cells and PCa cell lines. Clinical samples were used to confirm its expression. Investigations were then carried out *in vitro* and *in vivo* to determine the underlying mechanism of distinct circRNA on PCa metastasis. Advancements have been made in the contribution of circRNA to PCa progression as evidenced by the findings in **Table 3**. Recent research has focused on the regulation of circRNA expression in PCa, their potential value as biomarkers, their functioning in oncogenesis and treatment resistance (47).

**Table 3:** Aberrantly expressed circRNA regulate bone metastasis in PCa.

Circ RNAs	Expression in Tumour	Regulation mechanism	Related pathway	Experiment System	Ref.
circPDE5A	Downregulated	circPDE5A inactivates the MAPK pathway by interfering with EIF3C translation.	MAPK signaling pathway	*In vitro* and *in vivo* studies	L. Ding et al. (96) 2022
circ_0006156	Downregulated	circ_0006156 inhibits bone metastasis of PCa by targeting S100A9	S100A9	Clinical sample analysis, *in vitro* studies	Y. Zhang et al. (97) 2022
circDHRS3	Downregulated	miRNA sponge	miR-421/MEIS2	Clinical sample analysis, *in vitro* and *in vivo* studies	X. Dai et al. (98) 2023
circ_KATNAL1	Downregulated	miRNA sponge	miR-145-3p/WISP1	*In vitro* study	Y. Zheng et al. (99) 2020
circMID1	Upregulated	miRNA sponge	miR-330-3p/YTHDC2/IGF1R/AKT	Clinical sample analysis, *in vitro* and *in vivo* studies	Y. Ding et al. (100) 2022
circ_0063329	Downregulated	miRNA sponge	miR-605-5p/tgif2	Bioinformatics mining, *in vitro* and *in vivo* research	D. Lv et al. (101) 2023
circSCAF8	Upregulated	miRNA sponge	mir-140-3p/miR-335	Clinical sample analysis, *in vitro* and *in vivo* studies	T. He et al. (102) 2022
circEPHA3	Downregulated	miRNA sponge	miR-513a-3p/BMP2	*In vitro* and *in vivo* studies	H. Feng et al. (103) 2023
circ_SLC19A1	upregulated	miRNA sponge	miR-497/septin 2	Clinical sample analysis, *in vitro* study	Y. Zheng et al. (104) 2020
circ_0062019	Upregulated	miRNA sponge	miR-195-5p/HMGA2	*In vitro* study	P. Wang et al. (105) 2021
circSMARCA5	Upregulated	miRNA sponge	miR-432/PDCD10	*In vivo* study	C. Dong et al. (106) 2021

### Promotion of PCa bone metastasis

**Figure 5:**
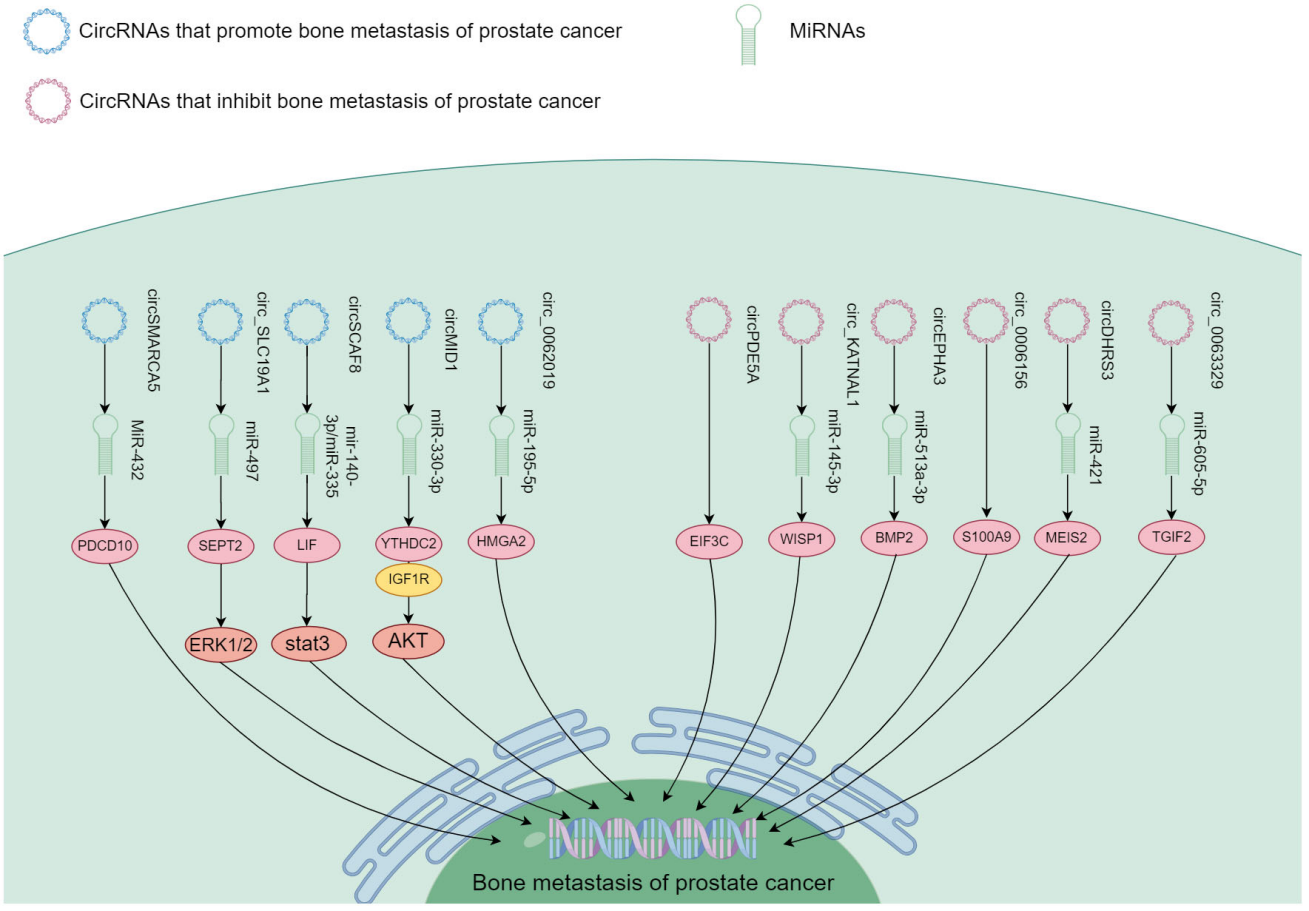
The mechanism of different circRNA responsible in PCa bone metastasis. These circRNA function in promoting and inhibiting PCa bone metastasis through the downstream signaling.

circRNA positively regulated PCa bone metastasis by inhibiting downstream miRNA through binding interaction (I don’t quite understand the precise meaning of spongification in this context, if you mean sopping up). Major circRNA/miRNA found to date include circMID1/miR-330-3p (100), circSCAF/miR-140-3p and miR-335 (102), circ_SLC19A1/miR-497 (104), hsa_circ_0062019/miR-195-5p (105), and circSMARCA5/MiR-432 (106). These circRNA were highly expressed in both PCa and bone metastasis, and functioned to promote bone metastasis through downstream signaling axes (**Figure 5**).

Representative mechanism of action was found for circMID1, which could bind miR-330-3p, and miR-330-3p could associate with IGF1R and YTHDC2. Functional experiments showed that circMID1/miR-330-3p regulated PCa progression through the YTHDC2/IGF1R/AKT axis (100). circ_SLC19A1 was found increased in PCa cells as well as the EV released by them. EVs with circ_SLC19A1 could be taken up by other PCa cells to promote cell proliferation and invasion. The expression of its target gene septin 2 (SEPT2) was significantly up-regulated. Hence, the expression of SEPT2 in cancer cells could be controlled through absorption of mir-497 by circ_SLC19A1 affecting the activation of the downstream ERK1/2 pathway, and ultimately growth and invasiveness of PCa cells (104). Another species, hsa_circ_0062019 enhanced HMGA2 expression by absorbing miR-195-5p. As a result, it stimulated PCa cells (105). Thus, a significant number of circRNA could function in the spread of PCa to bone.

### Inhibition of PCa bone metastasis

Some circRNA appeared to contribute to suppression of PCa bone metastasis. These include circDHRS3/miR-421 (98), circ_KATNAL1/miR-145-3p (99), hsa_circ_0063329/miR-605-5p (101), and circEPHA3/miR-513a-3p (103). These circRNA exhibited reduced expression in both PCa and bone metastasis. They suppressed PCa metastasis to the bone through downstream signaling pathways shown in **Figure 5**.

circDHRS3 regulated the expression of MEIS2 through circDHRS3/miR-421/MEIS2. *In vivo* experiments confirmed that overexpression of circDHRS3 could inhibit lung and bone metastases of PCa cells. circDHRS3 inhibited the proliferation and metastasis of PCa cells through circDHRS3/miR-421/MEIS2 (98);circ_KATNAL1 could bind to miR-145-3p, which could target WISP1 highly expressed in many tumor types. Circ_KATNAL1 and miR-145-3p promoted their own expression and down-regulated the expression of WISP1 (99). Thus, circ_KATNAL1 exerted a cancer-fighting function in PCa via miR-145-3p/WISP1. Functional experiments conducted *in vitro* and *in vivo* demonstrated that upregulation of hsa_circ_0063329 impeded the progression of PCa cells, whereas its suppression had the opposite effect (101). In high-grade PCa and cell lines, the expression of circEPHA3 was reduced. Its role as a tumor suppressor was demonstrated through its inhibition of PCa progression and metastasis. This was achieved by direct interaction of circEPHA3 with miR-513-a-3p/BMP2 to regulate downstream genes (103). Zhang et al. (97) found that circ_0006156 impeded PCa progression by binding to S100A9. *In vivo* studies demonstrated that it suppressed the motility and infiltration of PCa cells by enhancing expression of S100A9. Ding et al. (96) found that circPDE5A expression was down-regulated in PCa. This circRNA blocked WTAP-dependent N6-methyladenine(m6A) methylation of eukaryotic translation initiation factor 3c (EIF3C) by forming a circpDE5A-WTAP complex, which interfered translation to inactivate the MAPK pathway, thereby inhibiting PCa progression. Although these ncRNA showed low expression in cancer, circRNA might also play an inhibitory role in bone metastasis.

## Discussion

Considering the prevalence of PCa in men and the ongoing rise in incidence annually, it becomes more critical to devise novel screening techniques and therapies to supplement current ones. ncRNA are involved in gene expression, and are important in many signaling pathways. They regulate PCa bone metastasis, and can both promote and inhibit this process, which has been investigated in numerous studies. The findings indicate potential therapeutic and diagnostic development for PCa and bone metastasis. Their role still needs to be more thoroughly researched, and tested in clinical trials.

Abnormal miRNA expression plays a role in the onset of numerous cancer types (107). miRNA-associated signaling pathways could offer innovative approaches for treatment development. Takeshita et al. (108) demonstrated that miR-16 could be efficiently administered to PCa cells through tail vein injection in a mouse model of bone metastasis. A method for specific delivery of miRNA to target organs has, however, not yet been tested in the clinics. Importantly, miRNA could target numerous molecules, which poses a challenge in mitigating off-target effects. The same miRNA could have opposite effects in different tissues. Therefore, the total effect of miRNA-based treatments must be considered. The initial clinical testing involving liposome mimics of miR-34a for miRNA replacement therapy was ended early in two Phase I trials due to severe immune-related adverse events (109, 110). When the dosage of miR-16 mimics was lowered by approximately a thousand fold compared to that of miR-34a, no significant cytotoxicity was observed (110). Although this study particular showed acceptable safety and activity, additional trials are a must.

lncRNA are involved in gene expression as well, and important in the regulation of various signaling pathways. Abnormal lncRNA expression has been observed in different cancer types, including PCa (24). These observations suggested that they are a contributory factor in all stages of oncogenesis, PCa screening, diagnosis, prognosis and treatment. Currently, PCa suffers from overdiagnosis (111), and lncRNAs could become novel biomarkers in overcoming this problem.

circRNA are characterized by abundant, highly stable, evolutionarily conserved and tissue-specific expression. They play a part in oncogenesis, tumor spread, and treatment resistance (46). To date, tumor diagnosis and prognostic monitoring have largely relied on imaging and pathology. Currently, circRNA is employed as a biomarker in the identification and monitoring of urinary system tumors for diagnosis and prognosis. For example, circEGLN3 can distinguish renal clear cell carcinoma from normal tissue with 97 percent accuracy (112). Hence, circRNA could possess possibilities in the detection and surveillance of PCa bone metastasis. Furthermore, chimeric RNA could be another important type of ncRNA (113), and could influence the migration/invasion of PCa (114) although relevant studies are lacking.

Our current research suggests importance of ncRNA in PCa spread to bone. Their molecular mechanisms in cancer progression involves signaling pathways such as those of NF-κB, Wnt, TGF-β, PI3K/AKT, MMP and chemokines. Dysregulation of ncRNA may, however, occur due to other diseases or comorbidities. Further research is needed to determine the specific ones unique to PCa, if they exist. ncRNA are known to have multiple regulatory functions, and many are certainly not limited to PCa. Their action on normal human physiology needs to be fully explored to identify potential adverse side effects. At present, research on ncRNA employs mostly cell lines and animal models. Only when their functioning is thoroughly worked out can they be evaluated in clinical trials.

This review has some limitations. It only systematically summarized relevant studies in a 10 year time span. Due to the rather small number of confirmatory studies, quality could not be strictly evaluated.

## Conclusions

In summary, ncRNA could promote as well as inhibit PCa bone metastasis through multiple signaling pathways. Therefore, ncRNA are expected to be used as therapeutic targets for PCa. Their specific mechanism of action in bone metastasis still needs to be explored. Research on their clinical application is minimal. With further research, ncRNA will gain importance in treatment and patient monitoring.

## Author contributions

HS: Writing – original draft, Writing – review & editing. LL: Writing – original draft, Writing – review & editing. QZ: Writing – review & editing. YL: Writing – review & editing. JH: Writing – review & editing. PN: Writing – review & editing. ZH: Writing – original draft, Writing – review & editing. KC: Writing – original draft, Writing – review & editing.
